# Perspective taking: building a neurocognitive framework for integrating the “social” and the “spatial”

**DOI:** 10.3389/fnhum.2014.00403

**Published:** 2014-06-11

**Authors:** Antonia F. de C. Hamilton, Klaus Kessler, Sarah H. Creem-Regehr

**Affiliations:** ^1^Institute of Cognitive Neuroscience, University College LondonLondon, UK; ^2^Aston Brain Centre, School of Life and Health Sciences, Aston UniversityBirmingham, UK; ^3^Department of Psychology, University of UtahSalt Lake City, UT, USA

**Keywords:** social cognition, spatial cognition, social neuroscience, perspective taking, self-other distinction

From carrying a table to pointing at the moon, interacting with other people involves spatial awareness of one's own body and the other's body and viewpoint. In the past, social cognition has often focused on tasks like belief reasoning, which is abstracted away from spatial and bodily representations. There is also a strong tradition of work on spatial and object representation which does not consider social interactions. The 24 papers in this research topic represent the growing body of work which links the spatial and the social. The diversity of methods and approaches used here reveal that this is a vibrant and growing research area which can tell us more than the study of either topic in isolation.

Online mental transformations of spatial representations are often believed to rely on action simulation and other “embodied” processing and three papers in the current research topic provide new evidence for this process. Surtees and colleagues reveal that embodied egocentric transformations are used for visual as well as for spatial perspective taking, extending the generality of the embodied processing principle (Surtees et al., 2013). Braithwaite et al.'s contribution distinguishes between embodied and disembodied body-related hallucinations, showing that only the latter speeds up perspective taking (Braithwaite et al., 2013). Gardner and colleagues also highlight distinct processing routes towards perspective taking outcomes, where some individuals use embodied- while others use abstract (unembodied) calculation strategies (Gardner et al., 2013).

Several of the papers in this research topic have a focus on action systems in perspective taking. Creem-Regehr et al. analyze the literature on human judgments of other's affordances and how this relates to spatial perspective taking, concluding that these are complementary processes that work to inform understanding of another's behavior (Creem-Regehr et al., 2013). Maguinness et al. look at how observing another's action of lifting influences the discrimination of the weight of the objects lifted, and how this is modulated by age (Maguinness et al., 2013). Pezzulo et al. propose that that sensorimotor representations are recalibrated in social contexts to create shared action spaces serving joint action or more generally, social interaction (Pezzulo et al., 2013). Furlanetto et al. present a study examining the role of both gaze and action on perspective taking, finding the intriguing result that when gaze and action intention conflict, spontaneous perspective taking is increased (Furlanetto et al., 2013). Together, these papers suggest that perception, action and spatial processing all interact with and contribute to social cognition.

Direct interactions between spatial factors and social factors can be seen in a variety of domains, including emotional stimuli such as threat and pain. Takahashi et al. use virtual reality to show that potentially threatening objects are perceived as closer to the participant (Takahashi et al., 2013). Clements-Stephens et al. investigate the influence of the presence of an agent and the role of social skills on spatial perspective taking, finding a complex relationship among tasks, targets, and context (Clements-Stephens et al., 2013). Finally, the impact of perspective taking on observation of other's pain is examined by Canizales et al, finding both subjective evaluation and neural somatosensory responses are modulated by the perspective taken (Canizales et al., 2013).

The relevance of social and visuospatial perspective taking for successful communication is emphasized in five contributions in this research topic. Focusing on the integration of action- and spatial- perspective taking, Beveridge and Pickering propose that alignment of spatial perspectives may serve as a prerequisite for action language simulations (Beveridge and Pickering, 2013), in which language users adopt a particular action-perspective or frame-of-reference (FOR). Johannsen and De Ruiter show that priming of a relative FOR can dominate an a priori preference for an intrinsic FOR in communication, while communicative success is predicted by the amount to which interlocutors adapt to each other's strategies—whatever these are (Johannsen and Ruiter, 2013). De Boer and colleagues approach the question of communicative success from the angle of individual traits and report that motivational as well general-purpose cognitive abilities play a crucial role (De Boer et al., 2013). The flexibility of perspective taking in communication is further highlighted by Galati and Avraamides who show that people weigh multiple cues (including social ones) to consider the relative difficulty of perspective-taking for each partner, and adapt behavior to minimize collective effort (Galati and Avraamides, 2013). In this context cultural background could make a difference. Wu and colleagues report that Westerners and East-Asians differ in their strategies of controlling ego- vs. other-centred perspective taking outcomes but are similar in their immediate (egocentric) integration of communication context (Wu et al., 2013).

Developmental and neuroscientific approaches are also important in understanding perspective taking. New data from Hirai and colleagues shows that people with William syndrome find it hard to perform a level 2 visual perspective taking (VPT2) task, and this may be due to difficulties in spatial processing of body postures (Hirai et al., 2013). These data complement the review from Pearson et al. which shows that children with autism also find these VPT2 tasks hard (Pearson et al., 2013). Though Williams syndrome and autism are sometimes considered to have opposite effects on social cognition, here the intersection of spatial and social processing seems to be difficult for both populations. Moll et al. argue against the traditional view that VPT is simpler than cognitive perspective taking (theory of mind) and suggest that social coordination and communication occurs developmentally prior to full VPT abilities (Moll and Kadipasaoglu, 2013). This view contrasts with the paper from Wheatley and colleagues which suggests that in human evolution, brain systems for spatial processing have been repurposed for social cognition (Parkinson and Wheatley, 2013). Finally, Schurz and colleagues report a meta-analysis of fMRI data showing that perspective taking and theory of mind engage overlapping brain regions (Schurz et al., 2013). Together, these studies show clear links between spatial and social processing, and the question of which is “primary” may become an important debate in the future.

Finally, advances in our experimental data need to be interpreted in a solid theoretical framework. Several rival theories are available. Gross and Profitt make the claim that social connections can modulate participant's perception of space (Gross and Proffitt, 2013). Sun and Wang consider how both spatial and social problems can be conceptualized in terms of different frames of reference, and can be broken down to similar low-level components (Sun and Wang, 2014). May and Wendt evaluate theoretical accounts of perspective taking with a focus on two different tasks that require laterality judgments (May and Wendt, 2013). Limanowski and Blankenburg take a very different approach, providing an account of the experience of “self” in terms of the free energy principle that a brain functions to minimize surprise (Limanowski and Blankenburg, 2013).

Overall, the variety of papers in this research topic reflect the diversity and dynamism of the field. Recognition of the importance of studying spatial and social information processing in the same framework has come from many angles. Future studies can examine how these different types of task can scaffold each other and interact, possibly in an embodied fashion, to enable humans to cooperate and engage in a social space.

## Conflict of interest statement

The authors declare that the research was conducted in the absence of any commercial or financial relationships that could be construed as a potential conflict of interest.
